# Migratory Restlessness in an Equatorial Nonmigratory Bird

**DOI:** 10.1371/journal.pbio.0040110

**Published:** 2006-04-04

**Authors:** Barbara Helm, Eberhard Gwinner

**Affiliations:** **1**Max Planck Institute for Ornithology, Andechs, Germany; Lund UniversitySweden

## Abstract

The urge of captive birds to migrate manifests itself in seasonally occurring restlessness, termed “Zugunruhe.” Key insights into migration and an endogenous basis of behavior are based on Zugunruhe of migrants but have scarcely been tested in nonmigratory birds. We recorded Zugunruhe of African stonechats, small passerine birds that defend year-round territories and have diverged from northern migrants at least 1 million years ago. We demonstrate that Zugunruhe is a regular feature of their endogenous program, one that is precisely timed by photoperiod. These results extend ideas of programs for periodic movement to include nonmigratory birds. Such programs could be activated when movements become necessary, in line with observed fast changes and high flexibility of migration. Attention to Zugunruhe of resident birds promises new insights into diverse and dynamic migration systems and enhances predictions of avian responses to global change.

## Abstract 2

Recording migratory restlessness--Zugunruhe--in a resident African stonechat reveals that such activity occurs and is timed by photoperiod, and has implications for future research on migration and its evolution.

## Introduction

Zugunruhe, the urge of captive birds to migrate, manifests itself in seasonally occurring restlessness. Studies of Zugunruhe [ 1– 6] in migrant birds have contributed substantially to our understanding of the endogenous basis of migratory behavior. The fact that Zugunruhe develops spontaneously in time for migration led to the discovery of circannual (from *circa* meaning “around” and *annus* meaning “year”) programs in birds [ 1, 4, 5]. These endogenous programs are specific to migrant populations: amount and directionality of Zugunruhe in orientation cages corresponded in some cases roughly to migratory route and direction, possibly explaining the journeys of naive birds [ 4– 9]. Rapid modification of Zugunruhe in artificial selection experiments [ 8, 10, 11] has pointed to considerable genetic components to migratory traits and has been paradigmatic for microevolutionary adjustments. In light of these discoveries, attention has focused on Zugunruhe of migrants, implicitly assuming an absence in resident birds. However, the few available studies on resident populations suggest low-level Zugunruhe, for instance, in white-crowned sparrows *(Zonotrichia leucophrys),* Australian silvereyes *(Zosterops lateralis),* blackcaps *(Sylvia atricapilla),* and domestic Japanese quail *(Coturnix c. japonica)* [ 6, 10, 12– 14]. Such observations were either discounted as ancestral [ 12, 13] or classified as other behaviors, for instance, dispersal, nomadism, or song [ 6, 10, 12– 15]. Only one study to our knowledge examined residents in some detail, involving orientation experiments on white-crowned sparrows. The subjects showed oriented activity, as expected of Zugunruhe sensu strictu [ 13]. In view of these suggestive data and increasing evidence of flexible migration systems in response to global changes [ 7– 9, 16– 21], the possibility of migratory programs in residents merits reevaluation.

We tested for the presence of Zugunruhe in stonechats *(Saxicola torquata),* utilizing extensive knowledge of a north temperate migrant *(S. t. rubicola),* and an equatorial resident *(S. t. axillaris).* Stonechats are ideal subjects for the study of time programs [ 4, 5, 22– 26]. They display persistent circannual rhythms of molt and reproduction under constant conditions [ 26]. Stonechats migrate at night, so Zugunruhe can be conveniently measured as nocturnal activity [ 6, 15, 25]. Migrants show distinct Zugunruhe, timed by precise photoperiodic programs. At later hatching dates, stonechats commence Zugunruhe at successively younger ages, presumably to prepare for timely departure [ 25]. In contrast to northern obligatory migrants, color-ringed stonechats from equatorial Kenya defended their breeding territories throughout the year [ 22]. Genetic distances between the disjunctly distributed taxa are large [ 23, 24]. We therefore predicted that African stonechats display no Zugunruhe and possess no migratory time programs. The prediction was tested by comparing activity patterns of African stonechats to Zugunruhe of European migrants [ 25]. The presence of Zugunruhe in resident stonechats would alter views of migration, extending ideas that a readiness to move is common in birds [ 8, 9, 17, 18, 20]. The results of our study yield strong support for this conclusion.

## Results

Nocturnal activity of African stonechats changed with season and in all cases deviated significantly from white noise (Box-Ljung statistics: *p* < 0.001, except one case *p* < 0.05) [ 27]. Figure 1 illustrates distinct bouts of nocturnal activity of African stonechats kept under constant native day length. Since the birds received no seasonal cues, the repeated, spontaneous outburst of nocturnal activity indicates an underlying endogenous component [ 4, 5, 26]. Pronounced individual differences, which are typical for endogenous programs, further consolidate the independence of nocturnal activity from external cues. Period lengths could not be determined because one full annual cycle does not allow sufficient accuracy [ 4] and because Zugunruhe bouts of many birds occurred at irregular intervals. In contrast, under European day length, Zugunruhe was synchronized to the time of year. It occurred in fall and spring, at similar times but in lesser amounts than in European stonechats ( Figure 2) [ 25]. Zugunruhe was more pronounced in spring compared to autumn. Figure 3 illustrates the relationship of hatching date and timing of the onset of Zugunruhe under European day length. African and European stonechats showed similar responses to day length cues, initiating Zugunruhe at successively younger ages with later hatching date (slope ± SE = −0.92 ± 0.10, *F*_1,35_ = 81.06, *p* < 0.001; slope and intercept did not differ between taxa). Photoperiodic responses of the taxa did not differ in precision (Bartlett's test: χ _1_^2^ = 0.00; *p* = 0.989).

**Figure 1:**
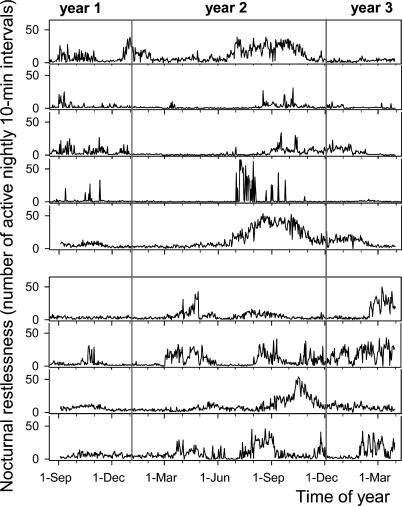
Nocturnal Activity under Constant Photoperiod (Light/Dark 12.25:11.75 h) Activity of five African stonechat males (upper panel) and four females (lower panel) recorded continuously in individual registration cages recorded for 1.5 years. Curves show nocturnal activity (Zugunruhe) as the number of 10-min intervals during which the birds were active at night. Since the birds received no temporal cues, the repeated, spontaneous outburst of nocturnal activity indicates an underlying endogenous component. Vertical gray bars indicate years.

**Figure 2:**
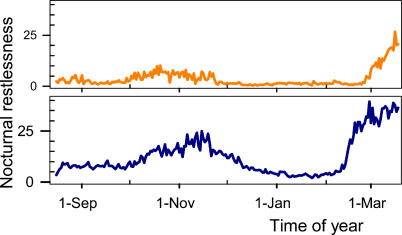
Zugunruhe under European Day Length Conditions Curves show mean nocturnal activity of ten resident African (upper panel, orange line) compared to 26 migrant European (lower panel, blue line) stonechats kept individually for one migration period under simulated natural European day length conditions; for details, see Figure 1.

**Figure 3:**
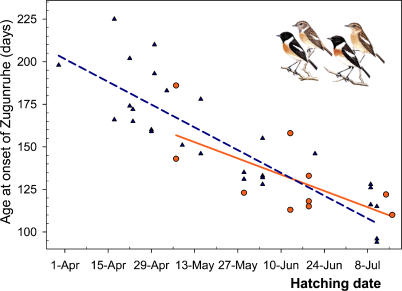
Relationship between Onset of Zugunruhe and Hatching Date African resident (orange dots, solid regression line; *n* = 10) and European migrant (blue triangles, dashed regression line; *n* = 26) stonechats initiated Zugunruhe at progressively younger ages when hatched late in the season under European day length conditions. Inlay: pairs of African (right) and European (left) stonechats; females are less conspicuously colored than males. Drawing is by H. Kacher.

## Discussion

Our data provide strong evidence for the presence of Zugunruhe in residents. Nocturnal activity of African stonechats was distinct and cannot easily be attributed to behaviors other than Zugunruhe. Its repeated, spontaneous occurrence under constant conditions suggests it to be part of an endogenous, probably circannual, program ( Figure 1), rather than a response to external cues [ 4, 5, 26]. The irregular time pattern of Zugunruhe under constant conditions is another common characteristic of resident and migratory stonechats (B. Helm, unpublished data). Under synchronizing light conditions, nocturnal activity coincided with spring and fall migration seasons ( Figure 2), as expected of Zugunruhe. Finally, the birds' responsiveness to differences in hatching date ( Figure 3) suggests that resident African stonechats, just like northern migrants [ 25], possess precise temporal programs for Zugunruhe. By advancing the onset of Zugunruhe, young stonechats largely compensated for differences in hatching date and started nocturnal activity at roughly the same time, as expected for seasonal movement [ 6, 25, 28].

Our study thus demonstrates common, complex features of Zugunruhe in resident and migrant birds, suggestive of ancestral patterns. However, Zugunruhe programs of African residents are unexpectedly precise, given an estimated divergence from a common ancestor 1 million to 3 million years ago [ 23, 24]. Furthermore, evolutionary rates and heritabilities of migratory traits are reportedly high [ 8, 11, 19]. This suggests that Zugunruhe programs of African residents may either be adaptive or maintained by stabilizing selection [ 29]. Assuming that Zugunruhe indicates time windows during which movements can easily be released or inhibited [ 1– 5], several selective advantages are conceivable. Persistent Zugunruhe windows could enhance and accelerate adjustments to changing conditions [ 8, 18, 20]. Intratropical movements are common in birds and could occur at times in African stonechat populations [ 9, 18, 23, 28, 30]. Southern African stonechat subspecies are thought to be partial altitudinal migrants [ 31]. The Kenyan population is distinct [ 24, 31] but also inhabits high altitudes. It is conceivable that periodically, altitudinal or other seasonal migrations, for example, related to drought, become necessary. Furthermore, the maintenance of Zugunruhe could be favored by related behaviors, for instance, dispersal [ 8, 17, 18, 20].

Based on our findings and a reassessment of literature evidence, we therefore propose that low-level Zugunruhe may be common in birds, including residents. Persistent migratory programs could underlie recent rapid changes in movement and range patterns attributed to global change and other human interventions [ 7– 9, 16– 20, 32]. This conjecture does not contradict theories of genetic influences on migratory traits [ 6– 11]. Rapid changes of Zugunruhe in breeding experiments could indicate selection on migratory thresholds or on traits related to expression of Zugunruhe [ 3, 6, 8– 11, 15, 32, 33]. The observed low level of Zugunruhe in some resident birds and its extinction by selective breeding do not imply that migration programs are absent. Locomotor activity has been shown to be heritable in rodent breeding experiments, as observed by Smith et al. (see page 437 in [ 13]) and recently elaborated by molecular genetics [ 34]. Underlying programs for seasonal movements may therefore be independent of changes in the expression of nocturnal activity.

We propose that the study of Zugunruhe of residents holds great promise for migration research. The success of such investigations depends on careful choice of study species. The most promising candidates include birds without known migratory relatives and taxa in which the evolution of migratory behavior has been conclusively resolved (K. Able, personal communication). New technologies promise exciting additional insights: image analyses of activities [ 35, 36], as well as recording of birds under more natural conditions with data loggers, open new possibilities to study migratory behavior in context. Such studies could also clarify links between activities like nomadism, dispersal, and migration [1–3,5–10,12–20,28,30,35] and ultimately allow another breakthrough in the understanding of avian movements.

## Materials and Methods

Offspring of stonechats from equatorial Kenya (0° 14′ S, 36° 0′ E) [ 22] were bred in our institute in Germany. At an age of 5 d, young were taken from the nests, hand-raised, and subjected to different light regimens (light intensities: 300 lux during the daytime and 0.01 lux during the nighttime) [ 26]. After independence, the birds were kept at an ambient temperature of 21 °C in individual registration cages. One experimental room stocked with 13 cages was exclusively used for African stonechats. The remaining birds were housed next to stonechats of other taxa. Temporal patterns showed that nocturnal activity was not synchronized among birds (e.g., Figure 1). One group of African stonechats was held for at least one migration period under their native constant photoperiod (15 birds; light/dark 12.25:11.75 h). Nine of these birds remained under constant conditions for 1.5 years ( Figure 1). Another group was monitored under the naturally fluctuating photoperiod of southern Germany (ten birds; 47.5° N) [ 25] and experienced winter day length of 40° N. Comparative data were derived from 26 migrant stonechats from Austria (48° 14′ N, 16° 22′ E) kept under identical conditions [ 25].

Nocturnal activity was recorded using passive infrared motion sensors. We recorded the number of movements within 10-min intervals and set a threshold to minimize background noise. A 10-min interval was scored as “active” if 20 or more movements were registered. Nocturnal activity was measured as the number of active 10-min intervals per night. Serial measurements of nocturnal activity were tested for deviation from white noise [ 8, 25]. To determine the onset of migratory activity, we applied a simple edge detector filter function [ 25, 27]. For each nocturnal activity value, the ten preceding and consecutive values were added with reversed signs. The resulting time series had clearly defined maxima indicating the onset of Zugunruhe.
